# *Heteropterys tomentosa* (A. Juss.) infusion counteracts Cyclosporin a side effects on the ventral prostate

**DOI:** 10.1186/1472-6882-13-30

**Published:** 2013-02-13

**Authors:** Karine M Freitas, Juliana C Monteiro, Marcos LM Gomes, Sebastião R Taboga, Heidi Dolder

**Affiliations:** 1 Department of Structural and Functional Biology, Institute of Biology, State University of Campinas (IB/UNICAMP), Campinas, SP, 13083-863, Brazil; 2 Department of Agrarian and Biological Sciences, CEUNES, Federal University of Espírito Santo, São Mateus, Brazil; 3 Department of Health Sciences, CEUNES, Federal University of Espírito Santo, São Mateus, Brazil; 4 Department of Biology, Institute of Biosciences, Language, and Science, State University of São Paulo - Júlio de Mesquita Filho, São José do Rio Preto, Brazil

**Keywords:** Cyclosporine, *Heteropterys aphrodisiaca*, Immunosuppressive drug, Medicinal plants, Phytotherapy, Male reproduction

## Abstract

**Background:**

Cyclosporin A (CsA) is an immunosuppressive drug widely used in treatment of auto-immune diseases or after organ transplants. However, several side effects are commonly associated with CsA long term intake, some regarding to loss of reproductive organ function due to oxidative damage. Considering that phytotherapy is an important tool often used against oxidative stress, we would like to describe the beneficial effects of *Heteropterys tomentosa* intake to minimize the damage caused by CsA to the ventral prostate tissue of Wistar rats under laboratorial conditions.

**Methods:**

Thirty adult Wistar rats (*Rattus norvegicus* albinus*)* were divided into: control group (water); CsA group (Cyclosporin A); Ht group (*H. tomentosa* infusion) and CsA + Ht group (CsA and *H. tomentosa* infusion). Plasmic levels of hepatotoxicity markers, triglycerides, cholesterol and glucose were quantified. The ventral prostate tissue was analyzed under light microscopy, using stereological, morphometrical and immunohistochemical techniques.

**Results:**

*H. tomentosa* did not cause any alterations either of the plasmic parameters or of the ventral prostate structure. CsA caused alterations of GOT, total and indirect bilirubin, cholesterol, triglycerides and glucose levels in the plasma; CsA-treated rats showed alterations of the ventral prostate tissue. There were no alterations regarding the plasma levels of GOT, triglycerides and glucose of CsA + Ht animals. The same group also showed normalization of most of the parameters analyzed on the ventral prostate tissue when compared to the CsA group. The treatments did not alter the pattern of AR expression or the apoptotic index of the ventral prostate epithelium.

**Conclusions:**

The results suggest a protective action of the *H. tomentosa* infusion against the side effects of CsA on the ventral prostate tissue, which could also be observed with plasmic biochemical parameters.

## Background

Cyclosporin A (CsA), which is a neutral, hydrophobic and cyclic peptide composed of 11 amino acid residues, was originally obtained from the fungi *Cylindrocapon lucidum* Booth and *Tolypocladium inflatum* Gams [1]. CsA is a powerful immunosuppressive drug that improves survival rates and decreases the rejection episodes and hospitalization days of transplanted patients [1,2]. CsA immunosuppressive properties are lymphocyte specific (cytotoxic and suppressor) and do not interfere with phagocyte functions or haemopoietic stem cells [2,3].

CsA treatment causes many collateral effects, with histopathological changes in various organs such as thymus, kidney, liver, heart, pancreas, brain [4], testis [5-10] and prostate [11]. The CsA-associated hepatotoxicity could be demonstrated by alterations of plasmic levels of glutamic pyruvate transaminase (GPT), glutamic oxalacetic transaminase (GOT), total proteins, albumin, bilirubin (total, direct and indirect) and total cholesterol [12-14]. In addition, the reduction of serum testosterone levels caused by CsA is reported by many authors [7,8,10].

Freitas et al. [11], working with Wistar rats, reported that CsA treatment reduced the ventral prostate weight, as well as the volume of prostate components (epithelium, lumen, muscular and non-muscular stroma), causing atrophy of the prostate secretory epithelium. According to the authors, these side effects would be associated both to the reduction of serum testosterone levels caused by CsA and to increased glycemia [11].

CsA use is fundamental in treatment of transplanted patients, as well as for those affected by autoimmune diseases. However, many side effects are related to its use, thus, many studies aim to discover substances that would decrease CsA side effects [6,12,15-19].

*Heteropterys tomentosa* A. Juss. (sin. *Heteropterys aphrodisiaca* O. Mach.) is a Brazilian native species; its roots are used by the local population as a tonic or stimulant in the treatment of the central nervous system debilities. Some studies also showed the aphrodisiac characteristic of this substance on male rodents [20-22]. A lyophilized extract of *H. tomentosa* has proven to inhibit the interaction among biomolecules and free radicals in the brain. Moreover, this treatment increased the amount of the antioxidant enzymes: SOD (Superoxide Dismutase), MnSOD (Manganese Superoxide Dismutase) and CuZnSOD (Copper-Zinc Superoxide Dismutase) in old rat brains [23], being efficient in the restoration of memory damage in these animals [24].

*H. tomentosa* is also known by its hypoglycemiant properties and has been used against *diabetes mellitus* by traditional communities in Mato Grosso state, Brazil [25,26].

Since *H. tomentosa* shows properties such as a strong antioxidant capacity [23,24], a hypolgycemiant effect [25,26] and an efficiency in reducing testicular damage caused by CsA intake (in Wistar rats) [6], we were motivated to analyze whether *H. tomentosa* infusion would be efficient in reducing the damage caused by CsA to the prostate tissue.

## Methods

### Medicinal plant and CsA

*Heteropterys tomentosa* roots were collected in Nova Xavantina (Mato Grosso, Brazil) and indentified by comparison with the voucher species of the plant in the Federal University of Mato Grosso herbarium, Brazil (registration number: 23928). The roots were dried at room temperature, being protected against rain and directed incidence of sunlight. After desiccation, they were crushed and powdered using a grinding mill. The infusion was prepared by pouring 100 ml of boiling water on 25 g of powdered roots. According to Marques et al. [27] water is the best extracting liquid for *H. tomentosa* roots regarding extractable solids. The final infusion had a yield of 6.832% (weight/weight) in terms of initial crude dry weight of plant material, producing 68.66 mg of extract dry weight per ml of infusion [6]. The methodology used to prepare the infusion and its dose were in accordance to that showed in previous studies [6,28-30]

Cyclosporin A (Sandimmun Neoral – Oral Solution; 100 mg/ml; Novartis Pharma AG, Switzerland) was diluted in distilled water or *H. tomentosa* infusion (dose of 15.0 mg/kg/day). CsA dose was chosen according to previous studies [6,11,31].

### Experimental animals and treatment protocol

*Rattus norvervegicus* albinus, Wistar rats, (24 animals, mean weight 405.66 g ± 29.76 g, 90 days-old) were randomly divided into 4 experimental groups (n = 6 in each group). The control group received distilled water; CsA group received Cyclosporin A; Ht group received *H. tomentosa* infusion; CsA + Ht group received CsA and *H. tomentosa* simultaneously. All treatments described above were administered daily by gavage (0.5 ml) during 56 days. CsA and *H. tomentosa* mixture was prepared diluting the dose of CsA in 0.5 ml of the plant infusion.

The animals were obtained from the Center for Biological Investigation - CEMIB (State University of Campinas, Campinas, SP, Brazil), and housed three per cage, under standard conditions with 12 hr. light/dark cycle.

Animals were weighted and anesthetized (between 9 to 11 o’clock in the morning) with xylazine and ketamine injection in the quadriceps femoris muscle (5 and 80 mg/kg body weight, respectively). The abdomen and thoracic cavities were opened. Testis, epididymis, seminal vesicle, coagulating gland, ventral prostate, kidney and liver were excised and weighed. The experimental protocol (#2295-1) was approved by the Institutional Committee for Animal Care and Use of the State University of Campinas.

### Blood samples and biochemical assays

Blood samples were taken from the left ventricle by cardiac puncture and stored in Vacuette® tubes (Sodium Heparin) under refrigeration (4ºC). They were centrifuged at 1,400x G, for 10 minutes (4ºC).

Blood samples were sent to specialized laboratories in order to quantify the amount of glutamic pyruvate transaminase (GPT), glutamic oxalacetic transaminase (GOT), total proteins, bilirubin (total, direct and indirect), triglycerides and cholesterol (all by enzymatic method); and albumin (by colorimetric method) (Laboratório Álvaro, Cascavel, Brazil), as well as glucose (by kinetic method) (Laboratório VetPat, Campinas, Brazil). These were quantified in order to detect possible toxic effect due to the plant infusion and/or CsA intake.

### Light microscopy

Following dissection, the ventral prostate lobes were separated; one of them was immersed in Karnovsky’s fixative (4% paraformaldehyde, 4% glutaraldehyde in 0.1 M phosphate buffer, pH 7.2) for 24 h at room temperature. After fixation, the tissue was routinely embedded in hydroxyethyl methacrylate (Historesin®, Leica). Two micrometer sections were obtained, stained with hematoxylin-eosin and observed with an Olympus BX-40 light microscope.

The contralateral lobe was immersed in Methacarn (Methanol: chloroform: acetic acid – 6:3:1) during 4 hours and paraffin embedded. Thus, 5 μm sections were made for AR immunohistochemistry and TUNEL assay.

### Morphometry and stereology

All morphometrical and stereological evaluations were made using the resin embedded material. The Image Pro Plus software was used for morphometrical and stereological analyses. The volumetric densities of lumen, epithelium, muscular and non-muscular stroma were determined using a 130-point grid system in ten randomly chosen microscopic fields. Absolute volumes were estimated multiplying the volumetric density by the prostate weight [32] since the ventral prostate density could be considered as approximately 1.0 [33]. The epithelial and muscular stroma thicknesses (μm) were measured. Nuclear area (μm^2^), perimeter (μm) and cytoplasm area (μm^2^) were measured and the form factor (4π X nuclear area / [nuclear perimeter]^2^) was calculated. For standardization, all quantifications were made of the intermediary region of the ventral prostate.

### AR immunohistochemistry

Paraffin was removed from sections prior to hydration. Antigenic retrieval was made by section immersion in citrate buffer, kept at high temperature (100°C) for 45 minutes. Hydrogen peroxide (3%) in methanol (during 20 minutes) was used to block endogenous peroxidase. The slides were incubated with the primary antibody anti-AR (Androgen receptor; SC-816, Santa Cruz Biotechnology, USA), at 37°C, for 1 hour and incubated with biotinylated secondary antibody at 37°C for 45 minutes. The slides were then incubated with peroxidase-conjugated avidin–biotin complexes. The positive reaction was revealed by diaminobenzidine. The sections were counterstained with Harris’s hematoxylin for 10 seconds.

### Terminal transferase d UTP Nick End Labeling (TUNEL) technique

A kit for fragmented DNA detection (US1QIA33-1EA; TdT FragEL TM DNA Fragment. Detect, Merck) was used for apoptotic nuclei detection. The slides were incubated with Proteinase K for permeabilization followed by endogenous peroxidase activity blocking with 3% hydrogen peroxide in methanol for 5 min. Equilibrium reaction, labeling, detection and revelation with diaminobenzidine were made according to the kit manufacturer’s instructions. The slides were counterstained with Harris’ hematoxylin for 8 sec.

The apoptotic index was calculated by counting labeled nuclei within the epithelium of 10 different fields (40x objective) for each animal, dividing the result by the total number of epithelial cells within the fields [34].

### Statistical analysis

Normality test was done for all the parameters, using the software Minitab 16. If the parameter values had normal distribution, the averages were compared by variance analysis (ANOVA) and with post hoc Duncan’s test, using the software Statistica 8. If not, the nonparametric test (Kruskal Wallis Multiple Comparison) was performed. For all the tests the significance was 95%. All data are presented as mean ± standard deviation (SD).

## Results

### Plasmic biochemistry assays

Plasmic levels of GPT, total proteins and albumin did not vary among experimental groups (Table 1).

**Table 1:** **Plasma biochemical dosages of Wistar rats treated with CsA, *H. tomentosa *infusion or both treatments simultaneously**

**Parameters**	**Experimental Groups**			
	**Control**	**CsA**	**Ht**	**CsA + Ht**
GOT (U/L)	**125.0 ± 33.05**^**a**^	**188.8 ± 39.8**^**b**^	**132.9 ± 53.32**^**ab**^	**127.6 ± 39.90**^**a**^
^#^GPT (U/L)	48.90 ± 8.59	73.36 ± 45.21	47.48 ± 10.14	58.42 ± 16.68
^#^Total Proteins (g/dL)	6.14 ± 0,089	6.08 ± 0.19	6.2 ± 0.141	6.12 ± 0.55
Albumin (g/dL)	3.64 ± 0.11	3.46 ± 0.18	3.58 ± 0.26	3.38 ± 0.15
Total Bilirubin (mg/dL)	**0.15 ± 0.04**^**a**^	**0.24 ± 0.01**^**b**^	**0.14 ± 0.031**^**a**^	**0.23 ± 0.04**^**b**^
Direct Bilirubin (mg/dL)	0.07 ± 0.04	0.05 ± 0.05	0.07 ± 0.03	0.08 ± 0.06
Indirect Bilirubin (mg/dL)	**0.08 ± 0.03**^**a**^	**0.20 ± 0.05**^**b**^	**0.07 ± 0.02**^**a**^	**0.15 ± 0.08**^**b**^
^#^Triglycerides (mg/dL)	**107.7 ± 44.74**^**a**^	**292.1 ± 74.10**^**b**^	**122.6 ± 78.07**^**a**^	**177.5 ± 94.88**^**ab**^
Cholesterol (mg/dL)	**62.74 ± 2.09**^**a**^	**73.16 ± 11.19**^**b**^	**59.48 ± 8.04**^**a**^	**79.18 ± 6.82**^**b**^
^#^Glucose (mg/dL)	**272.8 ± 27.58**^**a**^	**408.9 ± 65.17**^**b**^	**281.7 ± 44.83**^**a**^	**266.2 ± 48.45**^**a**^

Control: control group; CsA: Cyclosporin A treated group; Ht: *Heteropterys tomentosa* infusion treated group; and CsA + Ht: group with simultaneously treatment with CsA and *H. tomentosa*.

The values are mean ± SD. In each row, values with different superscripts are significantly different (p < 0.05). The parameters preceded by # did not show normal distribution, and therefore were compared using a nonparametric test. The others were compared with ANOVA *post hoc* with Duncan’s test.

GOT plasmic level was increased in the CsA group when compared to the control (p = 0.041). That parameter was normal for CsA + Ht group. Total bilirubin was significantly higher for the groups treated with CsA (CsA and CsA + Ht) when compared to the control (p = 0.0004 and 0.001, respectively). The dosage of indirect bilirubin showed a significant increase in the CsA and CsA + Ht groups (p = 0.003 and 0.045, respectively) (Table 1).

Triglycerides, cholesterol and glucose levels were significantly higher in the CsA group, when compared to the control (p = 0.006, 0.049 and 0.001, respectively) (Table 1). The increase of triglycerides and glucose levels caused by CsA treatment were not observed in the CsA + Ht group; however the increase of cholesterol levels was observed (p = 0.005).

The treatment with *H. tomentosa* infusion (Ht group) did not cause any alteration of the plasmic parameters analyzed.

### Biometrical parameters

**Figure 1:**
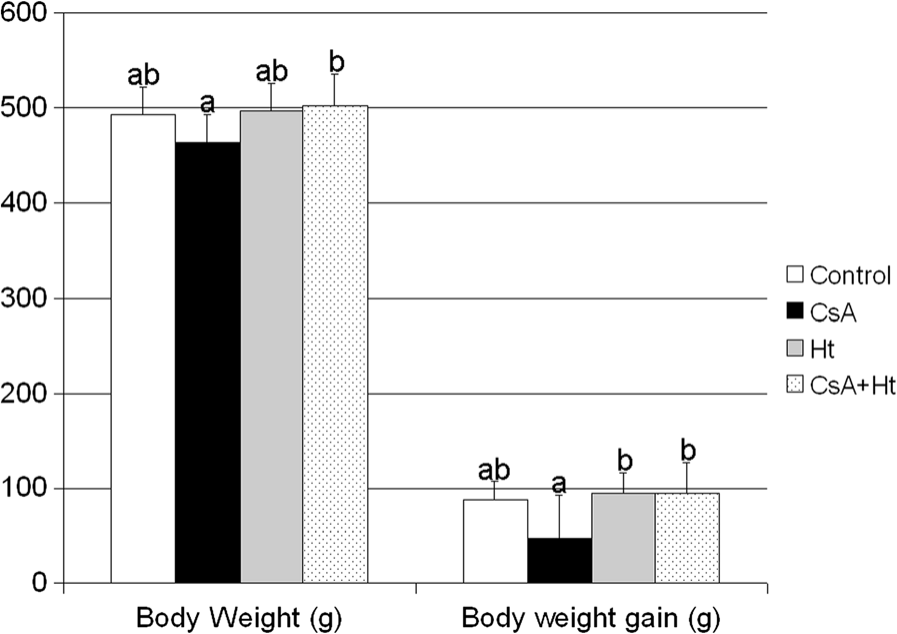
**Body weight and body weight gain (both in grams) of Wistar rats treated with water (Control), Cyclosporin A (CsA), *Heteropterys tomentosa *infusion (Ht), CsA and *H. tomentosa *infusion simultaneously (CsA + Ht).** The values are mean ± SD. In each column, values with different letters are significantly different (p < 0.05), according to Duncan’s test.

The treatment with CsA did not significantly alter the final body weight or body weight gain in CsA and CsA + Ht group (Figure 1). However the final body weight and body weight gain were lower in CsA group when compared to CsA + Ht group (p = 0.029 and 0.027, respectively) (Figure 1).

CsA treatment caused reduction of the ventral prostate (p = 0.006), coagulating gland (p = 0.002) and epididymis (p = 0.022) weights and increased kidney (p = 0.009) and liver (p = 0.016) weights in the CsA group when compared to the control (Table 2). CsA + Ht group did not show any alteration of the ventral prostate, epididymis, kidney and liver weights when compared to the control animals, although the coagulating gland weight was lower in this same group (p = 0.010) (Table 2).

**Table 2:** **Absolute (g) and relative (%) organs’ weight of Wistar rats treated with CsA, *H. tomentosa *infusion or both simultaneously**

**Parameters**	**Experimental Groups**			
	**Control**	**CsA**	**Ht**	**CsA + Ht**
*Weight (g)*				
Ventral Prostate	**0.499 ± 0.13**^**a**^	**0.332 ± 0.04**^**b**^	**0.481 ± 0.08**^**a**^	**0.398 ± 0.11**^**ab**^
^#^Seminal Vesicle	0.97 ± 0.10	0.79 ± 0.27	0.93 ± 0.27	0.82 ± 0.17
Coagulating Gland	**0.21 ± 0.03**^**a**^	**0.15 ± 0.02**^**b**^	**0.21 ± 0.04**^**a**^	**0.16 ± 0.03**^**b**^
Testis	1.96 ± 0.11	1.88 ± 0.23	1.97 ± 0.14	1.87 ± 0.19
Epididymis	**0.61 ± 0.02**^**ac**^	**0.55 ± 0.53**^**b**^	**0.64 ± 0.03**^**c**^	**0.58 ± 0.05**^**ab**^
Kidney	**1.49 ± 0.16**^**ac**^	**1.74 ± 0.17**^**b**^	**1.52 ± 0.13**^**c**^	**1.69 ± 0.17**^**bc**^
Liver	**15.92 ± 2.23**^**a**^	**19.25 ± 1.91**^**b**^	**15.49 ± 2.64**^**a**^	**16.80 ± 2.62**^**ab**^

Control: control group; CsA: Cyclosporin A treated group; Ht: *Heteropterys tomentosa* infusion treated group; and CsA + Ht: group with simultaneously treatment with CsA and *H. tomentosa*.

The values are mean ± SD. In each row, values with different superscripts are significantly different (p < 0.05). The parameters preceded by # did not show normal distribution, therefore were compared using a nonparametric test. The others were compared with ANOVA *post hoc* with Duncan’s test.

### Stereology and morphometry

The volumetric proportions of ventral prostate tissue components (lumen, epithelium, muscular and non-muscular stroma) did not vary among the experimental groups (Data not shown).

**Figure 2:**
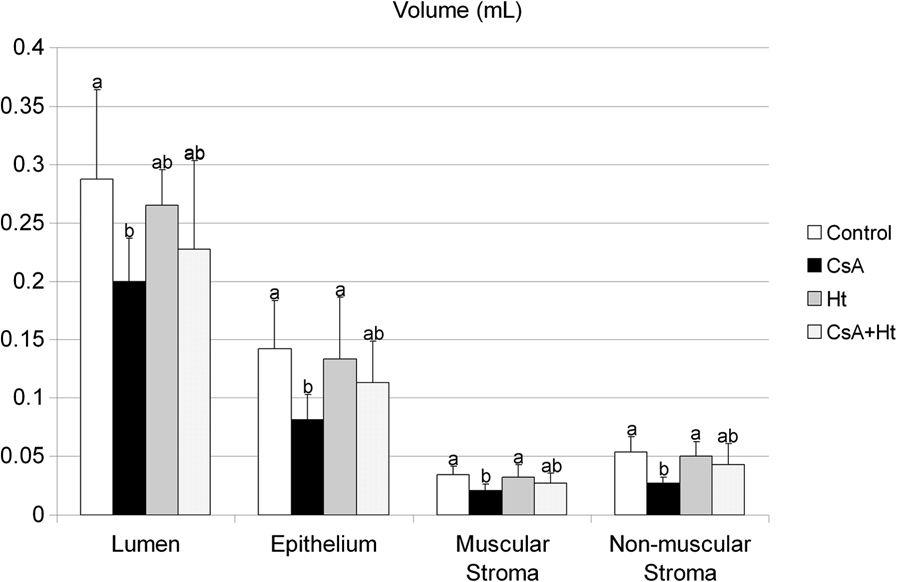
**Volume (mL) of ventral prostate tissue components (lumen, epithelium, muscular and non-muscular stroma) of Wistar rats treated with water (Control), Cyclosporin A (CsA), *Heteropterys tomentosa *infusion (Ht), CsA and *H. tomentosa *infusion simultaneously (CsA + Ht).** The values are mean ± SD. In each column, values with different letters are significantly different (p < 0.05), according to Duncan’s test.

CsA-treatment caused significant reduction of the volume of all ventral prostate tissue components - lumen, epithelium, muscular and non-muscular stroma - in the CsA group when compared to the control (p = 0.027, 0.022, 0.019 and 0.004, respectively) (Figure 2). However, when CsA was administered simultaneously with *H. tomentosa*, this reduction did not occur (Figure 2).

**Figure 3:**
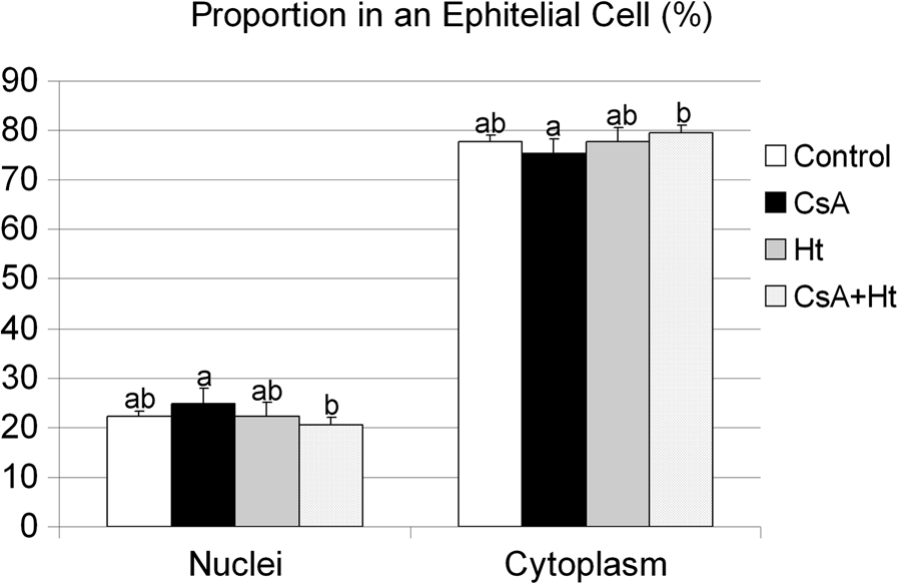
**Proportion between nucleus and cytoplasm of a ventral prostate epithelial cell from Wistar rats treated with water (Control), Cyclosporin A ( *CsA *), *Heteropterys tomentosa *infusion ( *Ht *), CsA and *H. tomentosa *infusion simultaneously ( *CsA + Ht *).** The values are mean ± SD. In each column, values with different letters are significantly different (p < 0.05), according to Duncan’s test.

**Figure 4:**
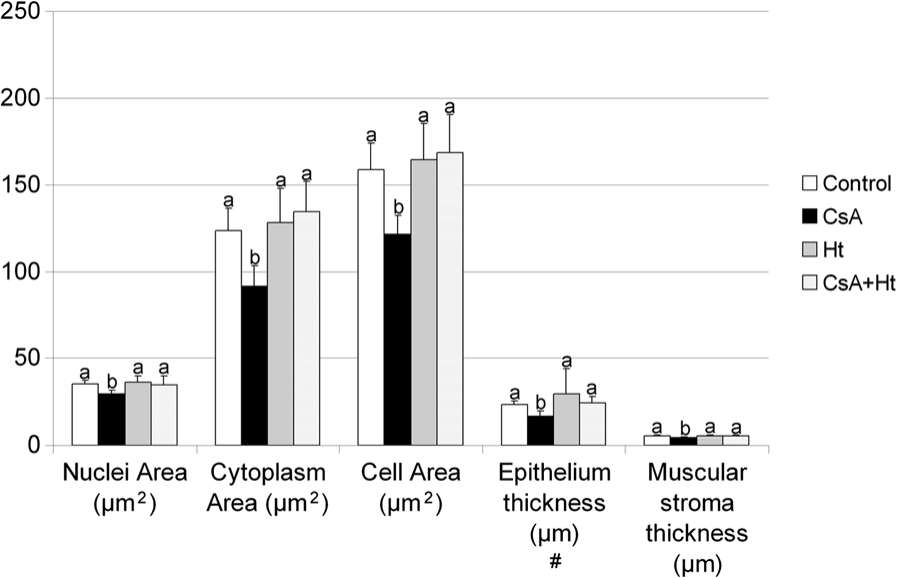
**Nuclei, cytoplasm and total epithelial cell areas; epithelium and muscular stroma thickness of the ventral prostate of Wistar rats treated with water (Control), Cyclosporin A (CsA), *Heteropterys tomentosa *infusion (Ht), CsA and *H. tomentosa *infusion simultaneously (CsA + Ht).** The values are mean ± SD. In each column, values with different letters are significantly different (p < 0.05), according to Duncan’s test, except for Epithelium thickness (#) - Kruskal Wallis Multiple Comparison test.

The proportion of nuclei and cytoplasm of the ventral prostate epithelial cells in the CsA group was higher or lower, respectively, when compared to the values of CsA + Ht group (p = 0.007 for both) (Figure 3). The total (p = 0.002), nuclear (p = 0.015) and cytoplasmatic (p = 0.002) area of ventral prostate epithelial cells was smaller in the CsA treated group when compared to the control (Figure 4). Similar result was not observed when the plant infusion was administered along with CsA (Figure 4).

The CsA group also showed reduced epithelium and muscular stroma thickness when compared to the control (p = 0.022 and 0.014, respectively). This result was not observed for the CsA + Ht group (Figure 4).

### AR immunohistochemistry and TUNEL

**Figure 5:**
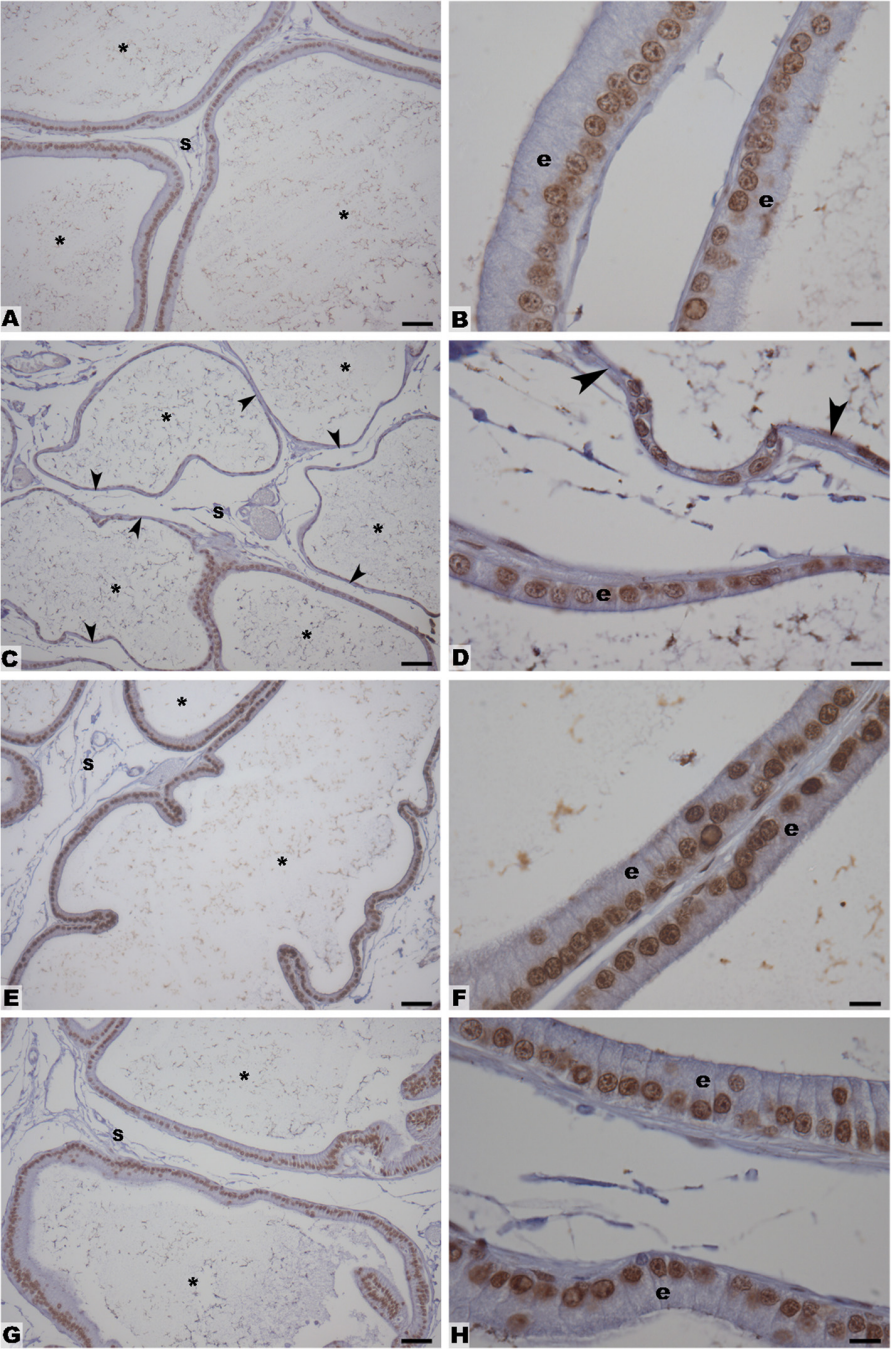
**AR immunohistochemistry in the ventral prostate sections. Groups: Control (A and B), CsA-treated (C and D), *H. tomentosa *infusion treated (E and F) and treated simultaneously with CsA and infusion (G and H).** Legend: arrowheads = atrophied epithelium; s = stroma; asterisks = lumen; e = epithelium. Bars = 50 μm (A, C, E and G) and Bars = 10 μm (B, D, F and H).

Most nuclei of all experimental groups were AR positively labeled (Figure 5). Some stromal cells also showed the same positive reaction (Figure 5). There were no differences in the pattern of AR expression among the experimental groups (Figure 5).

The apoptotic index did not vary after the treatments, being lower that 0.5% in all of them.

## Discussion

The CsA dose and the administration route employed in the present study were in accordance with the manufacturer’s recommendations for patients after organ transplantation and the same used in previous studies [6,16,18,35]. The method of *H. tomentosa* infusion preparation and the dose administrated was also based on methods applied in previous studies [6,28-30].

CsA administration is fundamental to avoid tissue rejection after organ transplantation and against auto-immune diseases, although several important side effects are reported. Thus, many studies seek a substance that would be able to diminish CsA side effects [6,12,16-19,35,36]. Many substances were efficient to protect the renal tissue of Wistar rats from CsA induced nephropathy, among them, Vitamin E [15], L-propionylcarnitine [36], carvedilol [16], green tea extract [17] and shallot (*Allium ascalonicum* L.) extract [19]. Furthermore, Kurus et al. [12] observed that oral administration of L-arginine prevented liver damage caused by CsA administration. Regarding the protection against CsA testicular impairment, Türk et al. [18] observed that lycopene had a potential protective effect against the CsA-induced oxidative stress leading to structural and functional damages in the testicular tissue and sperm quality of rats. Finally, Monteiro et al. [6] observed that *H. tomentosa* infusion was efficient to protect the testicular parenchyma, specially the seminiferous epithelium, against structural damage caused by CsA.

*Heteropterys tomentosa* infusion did not cause alteration of GOT, GPT, bilirubin (total, direct and indirect), total proteins, albumin, urea, cholesterol, triglycerides or glucose plasmatic levels, indicating that the plant infusion had no toxic effect.

In the present study, CsA did not cause alterations of the GPT levels, total proteins and albumin; but significantly increased GOT levels. According to previous studies [12,13], CsA-induced hepatotoxicity can be detected either by elevated serum GOT and GPT levels and/or reduced total protein content. Kurus et al. [12] working with animals treated with 7.5 mg/kg/day of CsA injected subcutaneously during 28 days observed increased levels of GOT and GPT. On the other hand, in the study carried out by Bohmer et al. [31] using CsA (5 and 15 mg/kg/day) administered by gavage during 8 weeks, the authors did not observe alterations of GOT and GPT levels. These authors had the same experimental protocol of that used in the present study, the differences in the results are probably due to individual differences of the experimental animals in relation to the treatment. Thus, we believe that the elevation of GOT level observed in the present study can be related to CsA hepatotoxicity; the group treated with *H. tomentosa* infusion along with CsA did not show altered GOT levels indicating attenuation of CsA-associated hepatotoxicity.

Increased total serum bilirubin is usually associated with CsA-induced hepatotoxicity [12,14]. In the present study, the increase of total plasma bilirubin observed in CsA and CsA + Ht groups was due to the increase of indirect bilirubin, since there was no alteration of direct bilirubin levels; this result could indicate that CsA affects the conversion of unconjugated bilirubin (indirect) to conjugated bilirubin (direct), probably due to hepatic damage.

The CsA-induced hepatotoxicity is probably related to the increase of reactive species of oxygen as confirmed by previous studies [12,37]. According to Rezzani et. al. [37] the responsive mechanism for oxidative stress associated with CsA administration is due to the imbalance between free oxygen radical production and the liver’s antioxidant defense system. CsA-induced oxidative stress is positively correlated with biochemical parameters characteristic of hepatotoxicity [13].

CsA treatment increased total cholesterol of both groups treated with the drug; this result was similar to that observed by Vaziri et al. [38]. Triglycerides and glucose levels were higher in the CsA group. Such higher levels were not observed in the animals that received the plant infusion along with CsA. Previous studies showed that hyperglycemia is associated with CsA-treatment [11,39]. *H. tomentosa* is popularly known as a hipoglycemiant medicinal plant [25]; the maintenance of normal glucose levels in the CsA + Ht group could be related to the hipoglycemiant property of *H. tomentosa*. The animals treated only with *H. tomentosa* showed no alteration of glucose levels. Therefore, *H. tomentosa* could be efficient as a hipoglycemiant only in animals with hyperglycemia.

The body weight was assessed to determine the effects of the CsA treatment on the general health of the animals [40]. Monteiro et al. [6] did not observe any significant difference of body weight gain of animals treated with CsA or CsA along with *H. tomentosa*, as was also observed in the present study. However, the final body weight and body weight gain increased in the group treated simultaneously with CsA and *H. tomentosa*, when compared to that treated only with CsA.

The ventral prostate and coagulating gland are androgen responsive glands and their weight variations could be related to testosterone level alterations [41,42]. The reduction of those glands weights could be related to testosterone level reduction caused by CsA. The CsA + Ht group also showed reduction of the coagulating gland, when compared to the control but the ventral prostate weight did not vary. Monteiro et al. [6] working with the same dose/treatment period of CsA and *H. tomentosa* did not observe alterations of ventral prostate and coagulating gland weights, contrary to that observed in the present study. Possibly this could be due to individual differences in the animals’ response and sensitivity to the treatment. Türk et al. [18] observed lower seminal vesicle weights in rats treated with CsA (dose of 15 mg/kg by subcutaneous injection, during 21 days) and unaltered seminal vesicle weights in rats treated with CsA + lycopene. These authors did not observe variation of testis, epididymis and prostate weights. The difference between the present research and the findings of Türk et al. [18] could be due to the difference in the CsA administration route and treatment duration.

CsA caused extensive damage in the ventral prostate tissue and atrophy of some epithelial regions [11]. Ultrastructural analyses showed that there was less rough endoplasmic reticulum and Golgi complex in atrophied cells, which suggests that these cells would have lower secretory activity. The damage caused by CsA to the prostate tissue was probably related to the lower serum testosterone levels and higher glucose levels [11].

The morphometrical and stereological analyses showed that simultaneous CsA and *H. tomentosa* administration protected the prostate tissue against the impairment caused by CsA administration. The alterations observed in the volume of prostate tissue components (lumen, epithelium, muscular and non muscular stroma) in the CsA group, were not observed in the CsA + Ht group. The ventral prostate epithelium and muscular stroma thicknesses, as well as the area of a ventral prostate epithelial cell (area of nucleus + cytoplasm) were significantly higher in the CsA + Ht group, when compared to the CsA group. These results showed that the effects of CsA on ventral prostate tissue were reduced in the animals treated with *H. tomentosa* and CsA.

In the previous report of Shabisgh et al. [34], there was virtually no apoptosis in the ventral prostate of control Wistar rats. The apoptotic index of all the groups in the present study was less that 0.5, which is considered normal for the ventral prostate epithelium of non treated animals [34]. Freitas et al. [11] suggested that the unaltered apoptotic index in animals treated with CsA for 56 days was due to the long term treatment. In castrated animals the apoptotic index reaches its peak at 72 hours after surgery. After this period, it starts declining, becoming normal after 7 days [34,43]. Therefore, the apoptotic index was not a good parameter to observe possible benefits of *H. tomentosa* treatment associated with CsA administration over 56 days.

Androgen receptors are present in most epithelial and smooth muscle cells (periductal and perivascular) and in some fibroblast cells of the rat ventral prostate [30]. AR expression in all experimental groups was similar to that cited above. Since the reaction intensity of AR-positive cells could be related to an increase in AR expression [44], the absence of any variation suggests that no alteration of AR expression occurred.

Mattei et al. [23] observed that the treatment with *H. tomentosa* extract increased the total activities of SOD, MnSOD and CuZnSOD in old rats’ brains. Thus, the protection provided by *H. tomentosa* against the prostatic damage induced by CsA intake would be related to antioxidant properties of *H. tomentosa.* In addition, considering that CsA causes hyperglycemia, leading to epithelial atrophy in the ventral prostate, the hypoglicemiant properties of *H. tomentosa* would be directly related to the reduction of CsA-associated side effects.

## Conclusions

This study did not show any toxicity related to *Heteropterys tomentosa* infusion administration based on the plasma content. Furthermore it indicated possible protection of *H. tomentosa* infusion on the prostate tissue against the damage caused by CsA. The alteration of hepatotoxic plasmic biochemical parameters also showed that the CsA-induced hepatotoxicity was reduced in the animals treated simultaneously with the plant infusion. New investigations are necessary to evaluate whether the plant infusion would interfere with the CsA immunosuppressant activity.

## Abbreviations

AR: Androgen receptor; CsA: Cyclosporin A or Cyclosporin A treated group; CsA + Ht: Group treated simultaneously with Cyclosporin A and *H. tomentosa*; CuZnSOD: Copper-Zinc Superoxide Dismutase; GOT: Glutamic oxalacetic transaminase; GPT: Glutamic pyruvate transaminase; Ht: *H. tomentosa* treated group; MnSOD: Manganese Superoxide Dismutase; SD: Standard deviation; SOD: Superoxide dismutase

## Competing interests

The authors declare that they have no competing interests.

## Authors’ contributions

KMF participated in all steps of the study development. JCM participated of animals’ treatment, of microscopic analyzes of the ventral prostate, and of writing the manuscript and its correction. MLMG participated of animal’s treatment and euthanasia, of prostate sample processing, carrying out TUNEL and AR immunohistochemistry and on manuscript redaction and correction. SRT suggested all the methodologies related to the ventral prostate tissue and showed them to KMF. HD participated in supervision of all steps of the study, of writing the manuscript and its correction. All authors read and approved the final manuscript.

## Pre-publication history

The pre-publication history for this paper can be accessed here:

http://www.biomedcentral.com/1472-6882/13/30/prepub
